# A Social Media-Based Acute Alcohol Consumption Behavior (NekNomination): Case Series in Italian Emergency Departments

**DOI:** 10.2196/ijmr.6573

**Published:** 2018-01-31

**Authors:** Stefania Barbieri, Paolo Feltracco, Vittorio Lucchetta, Rosa Maria Gaudio, Alberto Tredese, Mauro Bergamini, Gianna Vettore, Vincenzo Pietrantonio, Francesco Maria Avato, Daniele Donato, Deris Gianni Boemo, Maria Vittoria Nesoti, Rossella Snenghi

**Affiliations:** ^1^ Department of Urgent and Emergency Care University of Padova Padova Italy; ^2^ Forensic Medicine and Toxicology University of Ferrara Ferrara Italy; ^3^ Preventive Medicine and Risk Assessment University of Ferrara Ferrara Italy; ^4^ Forensic Medicine and Toxicology University of Padova Padova Italy; ^5^ Department of Directional Hospital Management Padova Hospital Padova Italy

**Keywords:** alcohol drinking, drinking behavior, underage drinking, binge drinking, alcoholic intoxication, adolescent, neknomination, binge drinking, alcoholic games, social network

## Abstract

**Background:**

NekNomination, also known as NekNominate, Neck and Nominate, or Neck Nomination, is a social network–based drinking game which is thought to have originated in Australia and spread all over the world between 2013 and 2014. Individuals record videos of themselves while rapidly drinking excessive quantities of alcoholic drinks (necking) and then nominate friends to outdo them within 24 hours; the videos are then posted on social media such as Facebook or YouTube. The consequences of this drinking game have been very dangerous; at least 5 people under age 30 years have died after drinking deadly cocktails, and many others have suffered from alcohol intoxication.

**Objective:**

The goal of the research is to evaluate data about clinically important acute alcohol intoxication among teenagers and young adults and inform and educate the general public, especially parents, teachers, and health workers, about the spreading craze of dangerous Internet-related behavior among today’s teenagers and young people up to the age of 23 years.

**Methods:**

Patients aged 15 to 23 years with acute alcohol intoxication who came to the emergency department (ED) of 2 major hospitals in Italy from January 1, 2011, to June 30, 2014, were included in this study. Data were retrieved from prehospital and intrahospital medical records and included personal information, methods of intoxication, triage color code, date and time of access to the ED, any relevant signs and symptoms, blood alcohol concentration, and diagnosis at discharge.

**Results:**

A total of 450 young patients (male 277/450, 61.5%, female 173/450, 38.5%; age 15 to 16 years 15/450, 3.3%, age 17 to 18 years 184/450, 40.9%, age 19 to 23 years 251/450, 55.8%) were recruited. The causes of intoxication were happy hour, binge drinking, NekNominate, eyeballing, other alcoholic games, or a mix of them. Happy hour was found to be more common among the older patients, whereas NekNominate accounted for almost half of the youngest group of hospitalizations. Eyeballing occurred in 1.6% (7/450) of cases; binge drinking and other alcoholic games caused 23.3% (105/450) and 23.8% (107/450) of hospitalizations, respectively. On admission, 44.2% (199/450) of patients were assigned a red or yellow color code requiring immediate medical attention; about 14% of them required additional medical assistance (after being in the ED) or hospitalization, some in semi-intensive care units.

**Conclusions:**

Our study shows that the increased numbers of hospitalizations due to alcohol intoxication in the adolescent age group, as a consequence of NekNominate or other drinking games, is alarming and represents a serious public health issue. The potential markers of improper use of social networks must be clearly identified, including categories at risk of alcohol abuse, in order to develop intervention and prevention strategies in terms of education and awareness, which may help in averting potentially fatal episodes.

## Introduction

### Background

Drinking games are thought to have originated thousands of years ago, when the ancient Romans played “ *la Passatella*,” a game in which the loser had to pay for the other players’ beer. These episodes very often ended up in brawls [1].

The common concept now is that drinking games are: (1) social drinking events, (2) played according to a set of rules specifying when and how much players should drink, (3) designed to promote the rapid consumption of large amounts of alcohol to facilitate drunkenness, and (4) involve the drinkers in performing cognitive and/or motor tasks [2].

Polizzotto et al [3] classified drinking games into 2 categories: competitive versus noncompetitive and skill-based versus chance-based. The authors noted that competitive games which do not require skill (like NekNominate) typically involve drinking the most or fastest in a short period of time and are therefore the most dangerous. These kinds of drinking games often involve external cues that dictate participants’ alcohol consumption (eg, media games such as drinking each time a character on television says a certain word or phrase).

Drinking games inevitably promote heavy alcohol consumption [2] and, compared with other high-risk drinking behaviors like prepartying (ie, drinking before going out to a social event or gathering [4]), drinking games are a unique high-risk activity because their rules are expressly designed to encourage drunkenness, leading to binge drinking (ie, 5 or more consecutive drinks within a 2-hour time-span on at least 1 day over the past 30 days [5]).

Since 1995, binge drinking has been increasing among teenagers aged 15 to 16 years in Europe [6] and accounts for more than 90% of alcohol consumed by 12- to 17-year-olds [7]. About 16.5% of males and 14% of females aged 12 to 20 years are binge drinkers, and many adolescents start to binge drink at very young ages [8]. Underage alcohol use in general contributes to the top 3 causes of mortality in this age group—injury, homicide, and suicide [9]—and is associated with other high-risk behaviors including suicide attempts, illicit drug use, sexual activity, increased number of sex partners, riding with a driver who has been drinking, and dating violence victimization [10]. In addition, very early drinking (under the age of 14 years) confers additional health risks, including a potential 4-fold increase in the likelihood of developing alcohol dependence [11].

Alternative drinking games practiced by young people have recently appeared. NekNominate is one of them, as well as “kings,” “flip cup,” “beer roulette,” “beer pool,” “tequila roulette,” “speed pennies,” “fuzzy duck,” and many others. “Butt chugging,” also known as “enema alcohol,” is the introduction of alcohol directly into the rectum and colon through the anus [12]; the recent increase in complications related to this drinking game is assumed to be due to alcohol abuse and the growing popularity of various erotic practices [13]. 

“Eyeballing” is performed by pouring alcoholic substances directly into the eyes; substances with high alcohol content are generally preferred, although cases of misuse of other substances (eg, cinnamon schnapps, 15% to 50% alcohol by volume [ABV]) have also been reported [14]. Drinking hand sanitizer consists of getting drunk by deliberately ingesting the amount of alcohol contained in hand disinfectants as a surrogate for drinkable alcohol [15]. 

Some of these practices are known; others have not been documented in medical journals or have only been discussed as case reports giving basic information [16]. The popularity of these behaviors is increasing [17] and poses a considerable challenge for emergency department (ED) physicians and workers [18].

Peer pressure is a well-known, influential fuel for excessive alcohol consumption, and Web-based social networks now offer a platform to expand the extent and impact of peer-to-peer coercion [19]. Mu et al [20] also demonstrated a significant dose-response relationship between Internet use and binge drinking, especially among adolescents.

NekNominate is a novel game that involves individuals posting videos of rapid alcohol consumption (“necking”) on social media sites and nominating an acquaintance to exceed their behavior within 24 hours. The named individual will then drink a larger quantity or a more extreme mixture with associated illegal, risky, embarrassing, or self-injurious behaviors. Other substances are often added to the alcohol and have included eggs, goldfish, insects, rodents, battery fluid, motor oil, de-icer, and human urine; associated behaviors have included driving, biking, skateboarding, and swimming while drinking [21]. What draws particular attention to this phenomenon is the fact that, although traditional extreme-consumption drinking games may also be fatal, they often occur in group settings with specific rules and more social control of excessive actions, something which is lacking in NekNominate. This current trend is particularly dangerous, given overt peer pressure with the potential for cyberbullying and online shaming if the nominee does not complete the dare.

Media reports have identified 5 NekNominate-related fatalities in the United Kingdom and Republic of Ireland, all occurring in February 2014 among young men aged 19 to 29 years; 4 from presumed acute alcohol poisoning and 1 from drowning after drinking [21]. However, it is difficult to assess the incidence of or resulting morbidity from this game; there are hundreds of NekNominate videos posted to various social media sites with unknown medical sequelae.

### Objectives

The objective of our research is to evaluate data about clinically relevant (ie, requiring hospitalization) acute alcohol intoxication episodes among teenagers and young adults, while focusing on the methods of reaching drunkenness (competitions, games, occasional acute consumption during parties, binge drinking, or recreational alcohol ingestion) by analyzing ED admissions. In addition, we want to inform and educate the general public, especially parents, teachers, and health workers, about the rapid spread of dangerous Internet-related behaviors among today’s teenagers.

## Methods

This research is a retrospective longitudinal observational single-center study conducted at two major hospitals in Italy. Young patients who accessed the EDs of these hospitals from January 1, 2011, to June 30, 2014, were included in this study. Inclusion criteria are aged 15 to 23 years, diagnosis of acute intoxication by blood test, and positive history of alcohol intoxication due to happy hour, binge drinking, Neknominate, eyeballing, other alcoholic games, or a mix of them, with or without drunkenness-related injuries. Patients who are younger than 15 years or older than 23 years, have no diagnosis of acute alcohol intoxication, or have a negative history for above-mentioned alcohol-related activities are excluded from the study.

Patients included in this study were asked (directly or through their relatives or friends at the ED) for personal information and methods of intoxication. Also recorded for the sample of selected patients were triage color-code (red=emergency requiring immediate attention—zero waiting minutes; yellow=urgent—10 minutes; green=less urgent—30 minutes; and white=nonurgent—60 minutes), the date and time of access to the ED, any relevant signs and symptoms, blood alcohol concentration (BAC, determined with an enzymatic dehydrogenase method and related to the state of consciousness), and discharge diagnosis. Patients were classified by gender (M/F) and age group (15 to 16 years, 17 to 18 years, and 19 to 23 years).

## Results

Considering the inclusion and exclusion criteria, 450 young patients who accessed the ED for acute alcohol intoxication due to specific drinking behaviors were recruited. The reasons for their intoxication were happy hour, binge drinking, NekNominate, eyeballing, other alcoholic games, or a mix of these. Among these patients, 15 (3.3%) were aged 15 to 16 years, 184 (40.9%) were 17 to 18 years, and 251 (55.8%) were 19 to 23 years; in total, there were 277 boys (61.5%) and 173 (38.5%) girls. The different distribution of alcoholic consumption classified by drinking behavior and age group in the general population is listed in Table 1.

Generally, young adults accessed the ED during the weekend at night, and alcohol-related hospitalizations therefore had an important effect on ED personnel, particularly on weekends. Conversely, Neknomination cases in our sample of adolescents accessed the ED during afternoons between Monday and Friday and during evening hours from Wednesday to Sunday. Half (226/450, 50.2%) of the patients were admitted between 12 AM and 8 AM.

None of the 15- to 16-year-old patients had alcohol intoxication while attending a happy hour event, but 30.4% (56/184) and 24.7% (62/251) of the 17- to 18- and 19- to 23-year-old groups, respectively, got drunk during happy hour. NekNominate-related hospitalizations totaled 5.8% (26/450) in the entire sample and represented almost half of the youngest group of hospitalizations. Eyeballing accounted for 1.6% (7/450) cases; binge drinking and other alcoholic games caused 23.3% (105/450) and 23.8% (107/450) of hospitalizations, respectively. A total of 53.2% (98/184) of the patients aged 19 to 23 years had been driving under the influence of alcohol during the late hours of the weekend.

**Table 1:** Distribution of alcohol consumption classified by drinking behavior and age group in the general population.

Behavior	Number by age group and sex						Total, n (%)	
	15-16 (n=15)		17-18 (n=184)		19-23 (n=251)		(N=450)	
	M (n=10)	F (n=5)	M (n=110)	F (n=74)	M (n=157)	F (n=94)		
Happy hour	0	0	32	24	41	21		118 (26.2)
Binge drinking	3	3	25	26	31	17		105 (23.3)
Neknomination	6	1	6	4	8	1		26 (5.8)
Other alcoholic games	1	1	23	6	40	36		107 (23.8)
Eyeballing	0	0	3	0	4	0		7 (1.6)
Mixed behaviors	0	0	21	14	33	19		87 (19.3)

BAC in the population varied between 1.5 and 2.4 g/L; 56% (252/450) of patients stated that they had consumed an excessive amount of alcohol in a short period of time. The most common signs and symptoms included heart palpitations (231/350, 66.0%), headache (119/215, 55.3%), diarrhea (45/102, 44.1%), nausea and vomiting (286/432, 66.2%), anxiety (2/7, 28.6%), problematic behavior (5/7, 71.4%), hallucinations (54/215, 25.1%), convulsions (5/225, 2.2%), coma (68/450, 15.1%), unconscious state (194/450, 43.1%), slow respiration (81/450, 18.0%), and rectal edema and severe acute colitis (1/10, 10.0%).

On hospital admission, 44.2% (199/450) of the patients were assigned a red or yellow color code, requiring immediate medical attention; 4.4% (20/450) were transferred to the short-term medical observation ward because they required continuous clinical evaluation, diagnostic testing, and appropriate treatment protocols; 3.3% (15/450) were admitted to a semi-intensive care unit; 80.4% (362/450) were discharged within 72 hours; 6.0% (27/450) were hospitalized; 2.0% (9/450) were spontaneously discharged; and 4.3% (18/450) refused hospitalization.

## Discussion

### Principal Findings

To our knowledge, this is the first study analyzing the clinical consequences of drinking games, with particular attention to NekNominate as a social media–based phenomenon. Some of these practices are already known; others have not been documented in medical journals or have only been discussed as case reports giving basic information. Drinking behaviors including the alcoholic games Neknomination and eyeballing are still not well recognized as public health problems, but increasing numbers of adolescents are admitted to EDs for acute alcoholic intoxication.

Our sample contained 450 young patients aged 15 to 23 years who accessed the ED after ingesting excessive amounts of alcohol in an unconventional and dangerous way. Most of them were between 19 and 23 years old and male. Of all patients, happy hour (a special offer, in which drinks are cheaper at a certain time of day) was the most common way of reaching severe drunkenness (26% of all ED accesses), followed by various alcoholic games, binge drinking, mixed behaviors, NekNomination and eyeballing. Happy hour only affected patients aged 17 to 23 years, indicating that this practice is not frequent among younger people. Conversely, NekNomination was the most common cause of alcohol intoxication among the youngest patients (aged 15 to 16 years), representing 47% of all hospitalizations in this age group. This figure adds to the dangerousness of this practice in view of the fact that the earlier alcohol abuse begins, the worse the consequences (eg, problematic alcohol use in later life and increased susceptibility to high-risk styles of drinking [22]). In addition, as a social media–based drinking game, this highlights the importance of peer pressure, which is particularly influential in younger adolescents who are less able to resist this type of coercion.

Alcoholic games in general have caused increasing numbers of hospitalizations directly proportional to patients’ ages: 13% in the 15- to 16-year-old group, 16% in those aged 17 to 18 years, and 30% in those aged 19 to 23 years, and this matches the most recent reviews about the correlation of age with drinking games [22]. Most of the older patients were hospitalized at night over weekends; conversely, younger adolescents who practiced NekNomination accessed the ED during working days in the afternoon, indicating different drinking patterns in the various age groups.

The abuse of alcoholic drinks is a significant source of risk for adolescents and is one of the primary causes of many accidental injuries, such as falls, accidents at home, and road accidents (38% of the patients aged 19 to 23 years were driving under the influence of alcohol, thus also representing a danger for others). A large proportion (44%) of patients admitted to the ED were assigned a red or yellow color code, meaning that they required immediate or almost immediate treatment; about 14% of them required additional medical assistance (after being in the ED) or hospitalization, sometimes in a semi-intensive care unit, representing a significant burden in terms of personnel requirements and costs for diagnosis and treatments. The most common presenting signs and symptoms were those typical of alcohol intoxication: heart palpitations (66%), headache (55%), diarrhea (44%), nausea and vomiting (66%), and problematic behavior (63%).

NekNominate seems to be an on-off phenomenon related to 2013-2014; nevertheless, Wombacher et al [23] wrote about it and its relation to social norms in June 2016. Although NekNomination is no longer shown in the media and is now not a search trend in Google or Twitter, this does not mean the practice has disappeared: modern video exchange methods, (eg, Snapchat or WhatsApp) are now more private than in the past and allow users to exchange videos between individuals or groups of selected friends. Thus, in addition to the above considerations, we decided to focus on NekNominate because of its peculiar aspects: (1) it was probably the first drinking game which spread enormously through social media websites, allowing all participants to confront anyone anywhere in the world and thus setting a milestone in dangerous Web-based challenges, (2) it belongs to the category of competitive games that do not require skill and are proven to be the most hazardous [3], (3) it has recently caused drastic consequences such as deaths and hospitalizations (as reported here), (4) the role of peer pressure is extremely significant, as people refusing to accept the challenge are potentially victims of cyberbullying and therefore tend to exaggerate their behaviors in order not to be excluded by their peers, and (5) literature investigating this high-risk practice is still not frequently available.

**Figure 1:**
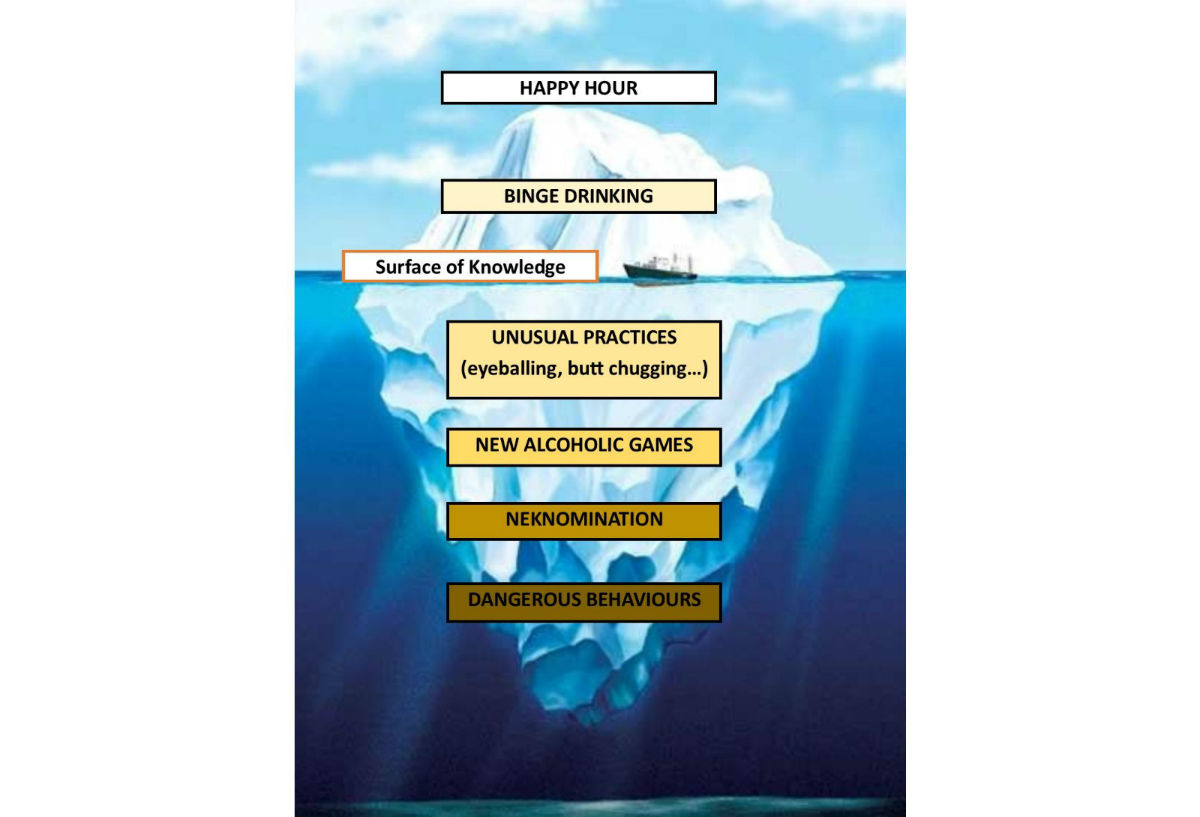
The iceberg of current knowledge of excessive drinking behaviors.

We present the various patterns of alcohol intoxication among adolescents with the image of an iceberg to highlight the magnitude of unknown behaviors compared with what is known in the scientific literature (Figure 1). There are many news reports on binge drinking and happy hour–related alcohol intoxication and alcohol (vodka) enema or eyeballing, especially concerning young males aged 24 to 27 years. Nevertheless, the scientific literature appears to be lacking in regard to these games, which are spreading at a dangerous rate and are rapidly affecting adolescents all over the world. 

Most of the iceberg is below the waterline and represents people’s unawareness regarding young people’s acute alcohol consumption. Binge drinking, Neknomination, and alcoholic games are also often associated with abnormal behaviors such as not wearing a helmet while riding a motorbike, not fastening a seatbelt while driving a car (both illegal in Italy/Europe), having promiscuous sex, and consuming illicit drugs.

The widespread use of social media is having a severe effect on our society: its easy accessibility makes it one of the most commonly used tools among teenagers and young adults, and the changes induced in current behaviors are still under evaluation [24]. Social media certainly played an important role in the spread of NekNominate; posting videos on the Web in order to collect likes and visualizations seems to be a way of getting into a group or being socially accepted by peers, and inviting friends to do the same has made the game spread rapidly among young people.

### Limitations

This study is limited by the small sample size, which is not representative of the entire Italian teenager population. However, it is still useful for preliminary data about drinking games.

### Conclusions

Drinking games are still relatively undisclosed and “at the bottom of the iceberg” of health workers’ attention and knowledge. They encourage young people to consume large quantities of alcohol within a short period of time, putting them at risk of alcohol intoxication that can potentially cause injuries, later regretted sexual activities, suicides, and traffic accidents and deaths.

Social media is an important tool for connections among young people and may be associated with behavioral and social maladjustment among adolescents. It also represents a powerful source of peer pressure. Adolescents who consume alcohol in new and dangerous ways such as NekNomination reach EDs with high BAC, are classified as red or yellow codes at triage, present severe impairment of vital functions, and often require further intensive medical assistance.

In order to develop a prevention strategy which could help to avert potentially fatal episodes, it is necessary to highlight the potential markers of problematic social network use and to inform and educate health personnel, social workers, and school teachers about these new phenomena, which are spreading very rapidly among adolescents. Further studies to better describe and analyze the problem, particularly in its clinical aspects, are in progress.

## Abbreviations

ABV: alcohol by volume
BAC: blood alcohol concentration
ED: emergency department
